# Anion Dependent Particle Size Control of Platinum Nanoparticles Synthesized in Ethylene Glycol

**DOI:** 10.3390/nano11082092

**Published:** 2021-08-18

**Authors:** Johanna Schröder, Sarah Neumann, Jonathan Quinson, Matthias Arenz, Sebastian Kunz

**Affiliations:** 1 Institute of Applied and Physical Chemistry (IAPC), Center for Environmental Research and Sustainable Technology, University of Bremen, Leobener Strasse 6, 28359 Bremen, Germany; SarahNeumann1@gmx.de; 2 Department of Chemistry, Biochemistry, and Pharmaceutical Sciences (DCBP), University of Bern, Freiestrasse 3, 3012 Bern, Switzerland; matthias.arenz@unibe.ch; 3 Chemistry Department, University of Copenhagen, Universitetsparken 5, 2100 Copenhagen, Denmark; jonathan.quinson@chem.ku.dk; 4 Südzucker AG, Central Department Research, Development, and Services (CRDS), Wormser Strasse 11, 67283 Obrigheim, Germany

**Keywords:** “surfactant-free” Pt nanoparticle synthesis, polyol process, anion dependent particle size control

## Abstract

The polyol synthesis is a well-established method to form so-called “surfactant-free” nanoparticles (NPs). In the present study, the NP size resulting from the thermal reduction of the precursors H_2_PtCl_6_, H_2_Pt(OH)_6_, or Pt(acac)_2_ in presence of the bases NaOH or Na(acac) at different concentrations is studied. It is shown that the size control depends more strongly on the nature of the precursor (metal salt) than on the anion present in the base. The latter is surprising as the concentration of the base anion is often an important factor to achieve a size control. The reduction of H_2_PtCl_6_ or H_2_Pt(OH)_6_ in presence of NaOH and Na(acac) confirm the observation that the NP size is determined by the OH^−^/Pt molar ratio and expands it to the base anion/Pt molar ratio. In contrast, the reduction of Pt(acac)_2_ in presence of the bases NaOH (previous reports) or Na(acac) (shown in the present work) leads to larger NPs of ca. 3 nm, independent of the concentration of the base anions. Hence, the anion effect observed here seems to originate predominantly from the nature of the precursor (precursor anion dependence) and only for certain precursors as H_2_PtCl_6_ or H_2_Pt(OH)_6_ the size control depends on the base anion/Pt molar ratio.

## 1. Introduction

**Figure 1:**
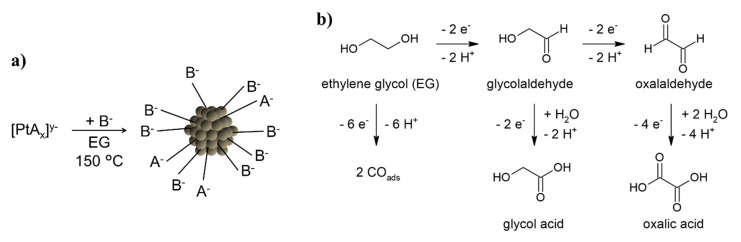
Scheme of the thermally induced polyol process: (**a**) the precursor with the anion A^−^ is reduced in presence of the base anion B^−^ in EG at 150 °C to form NPs. For instance, during the reduction of [Pt(OH)_6_]^2−^ the anions are A^−^ = B^−^ = OH; (**b**) different oxidation mechanisms of EG.

The polyol method performed in alkaline ethylene glycol (EG) is a versatile approach to synthesize “surfactant-free” Pt nanoparticle (NP) based tailored catalysts [1,2,3] for instance for CO oxidation [4,5] or fuel cell studies [6,7]. If desired, the “surfactant-free” particles can subsequently be functionalized with organic surfactants such as different amino acids, e.g., *L*-proline, to increase the catalytic activity [8] or to control the enantiomeric selectivity of hydrogenation reactions [9,10,11]. In the “surfactant-free” NP synthesis a Pt precursor (metal salt, e.g., H_2_PtCl_6_) is reduced in presence of a base like NaOH while the solvent EG is oxidized. The oxidation mechanisms of EG to form glycolaldehyde, glycol acid, oxalaldehyde, and oxalic acid have been described and additionally two CO molecules absorbing to the NP surface are formed [12,13]. The OH^−^ of the base is neutralized by the protons formed during the reduction of the metal precursor to form water, as shown in Figure 1 [14].

The influence of the anion OH^−^ as additive within the reaction mixture on the stability and size control was mentioned before in different studies [7,13,15]. Quinson et al. [16] investigated the influence of the cations Li^+^, Na^+^, K^+^, and Cs^+^ in the hydroxide base and showed that the particle size and stability is not affected in EG. Neumann et al. [4] observed that the presence of halides in the precursors H_2_PtCl_6_ and H_2_PtBr_6_ induces leaching leading to a size increase during the thermal reduction in presence of NaOH at 150 °C during 17 h. In contrast, the size of the halide free precursor Pt(acac)_2_ (acac− as abbreviation for the acetylacetonate anion) remains constant [4]. Due to the larger leaching ability of bromide as compared to chloride, the thermal reduction of H_2_PtBr_6_ (as compared to H_2_PtCl_6_) results in a larger particle size for prolonged reaction times. Furthermore, the use of the halide-free precursor Pt(acac)_2_ leads to substantially larger particles of about 3 nm [4]. Schröder et al. [15] showed that the use of the precursors H_2_PtCl_6_ or H_2_PtBr_6_ in presence of NaOH under conditions where Ostwald ripening can be excluded, i.e., reduction induced by visible light at room temperature, results in a comparable size control by the OH^−^/Pt ratio. The results of Neumann et al. [4] and Schröder et al. [15] indicate an influence of the precursor anions on the particle size. The size resulting of the reduction of the precursors H_2_PtCl_6_ [7,13,15] or Pt(acac)_6_ [4] in the presence of NaOH were investigated before.

In the present study, the precursors H_2_PtCl_6_ or Pt(acac)_2_, were reduced in presence of the base Na(acac), and H_2_Pt(OH)_6_ in presence of the bases NaOH or Na(acac). By doing so, the influence of the nature of the base anion of NaOH or Na(acac) and its concentration on the particle size in the reduction of the precursors H_2_PtCl_6_, H_2_Pt(OH)_6_, or Pt(acac)_2_ are compared. It is observed that, as mentioned by Quinson et al. [7], the size control by tuning the OH^−^/Pt ratio seems to be applicable to the base anion/Pt molar ratio for the precursors H_2_PtCl_6_ and H_2_Pt(OH)_6_. In contrast, a comparable large particle size of ca. 3 nm is obtained here in the reduction of Pt(acac)_2_ in presence of Na(acac) or in the reduction of Pt(acac)_2_ in presence of NaOH as shown by Neumann et al. [4]. This clearly shows the importance of the careful selection of the nature of the precursor (the precursor anion) compared to the nature of the base anion in order to achieve size control in ‘’surfactant-free’’ NP synthesis.

## 2. Experimental Section

### 2.1. NP Synthesis

In general, for the polyol process, a metal salt (precursor) is reduced in presence of a base in EG. The thermal reduction is performed in a preheated oil bath at 150 °C, see Figure 1 [4,7,13]. In the present study H_2_PtCl_6_, H_2_Pt(OH)_6_, or Pt(acac)_2_ were used as precursors and NaOH or Na(acac) as bases. Details about the syntheses are found in the following.

#### 2.1.1. Synthesis of “Surfactant-Free” Pt NPs by Thermal Reduction of H_2_PtCl_6_ in Presence of Na(acac)

H_2_PtCl_6_ xH_2_O (0.04 g, 40% Pt, Chempur, Karlsruhe, Germany, or AlphaAesar, Haverhill, MA, USA) was dissolved in 4 mL of EG (99.8%, VWR, Radnor, PA, USA) in a 50 mL round bottom flask. 4 mL of 0.5 M Na(acac) (C_5_H_7_NaO_2_ xH_2_O, 95%, AlphaAesar, Haverhill, MA, USA) in EG was added to obtain an acac^−^ starting concentration of 0.25 M, i.e., an acac^−^/Pt ratio of 24.4. Because of the impurities in the base (only a purity of 95%) proportionately more Na(acac) was dissolved in EG. During dissolution overnight, the solution turned light yellow. Different base concentrations between 0.055 and 0.5 M were obtained by diluting 0.5 M Na(acac) in EG leading to acac^−^/Pt ratios between 5.4 and 48.8. As the total volume of the added base was kept constant at 4 mL, the reaction mixture was in total 8 mL. The flasks were equipped with a reflux condenser. The mixture was vigorously stirred at 150 °C for 10 min using a preheated oil bath. The precursor solution turned dark brown after about 1–2 min indicating the NP formation. Previously unpublished results showed that at lower Na(acac) amounts the particles became instable even after keeping the synthesis temperature at 150 °C for 20 min. After the heating procedure, the reaction mixture was cooled to ambient temperature.

#### 2.1.2. Synthesis of “Surfactant-Free” Pt NPs by Thermal Reduction of H_2_Pt(OH)_6_ in Presence of NaOH or Na(acac)

For the reduction in presence of NaOH the precursor H_2_Pt(OH)_6_ (0.0286 g, 56% Pt, ChemPur, Karlsruhe, Germany) was dissolved in 4 mL of EG. To obtain the desired base starting concentrations the amount of OH^−^ in the precursor was considered and the 0.5 M NaOH (VWR, Radnor, PA, USA) stock solution in EG was diluted with EG to a total volume of 4 mL. The applied Pt concentration of 10.25 mM in the total volume of 8 mL led to an OH^−^ concentration of around 0.072 M for H_2_Pt(OH)_6_. Therefore, OH^−^ concentrations between 0.078 and 0.5 M were used for thermal reduction of H_2_Pt(OH)_6_, leading to OH^−^/Pt ratios of 7.6 to 48.8, respectively.

For the reduction in presence of Na(acac) the precursor H_2_Pt(OH)_6_ (0.0138 g, 57.94% Pt, ChemPur, Karlsruhe, Germany) was dissolved in 4 mL of EG. The 0.5 M Na(acac) stock solution was diluted by EG. Because of the impurity of the base (only a purity of 95%) proportionately more Na(acac) was dissolved in EG. During dissolution overnight, the solvent turned light yellow. For example, 4 mL of 0.5 M Na(acac) in EG was added to obtain an acac^−^ starting concentration of 0.25 M, leading to an acac^−^/Pt ratio of 48.8.

As the total volume of the added bases was kept constant at 4 mL, the volume of the reaction mixtures in both synthesis approaches with the two different bases was in total 8 mL. The flask was equipped with a reflux condenser. H_2_Pt(OH)_6_ is insoluble at room temperature in EG, it stays turbid even after one week of stirring in the dark. The solution and reduction step of H_2_Pt(OH)_6_ in EG could not be separated from each other as the white turbidity changed first to yellow and then brown turbidity before the brown dispersions were obtained by heating up. Therefore, the turbid mixture was vigorously stirred at 150 °C for 90 min using a preheated oil bath. The precursor solution of H_2_Pt(OH)_6_ in presence of NaOH turned dark brown after about 1 to 25 min (the lower the OH^−^ concentration, the faster the reduction) indicating NP formation. The precursor solution of H_2_Pt(OH)_6_ in presence of Na(acac) turned dark brown after about 90–120 s. After the heating procedure, the reaction mixture was cooled to ambient temperature.

#### 2.1.3. Synthesis of “Surfactant-Free” Pt NPs by Thermal Reduction of Pt(acac)_2_ in Presence of Na(acac)

Pt(acac)_2_ (0.0161 g, 49.6% Pt, FluoroChem, Hadfield, United Kingdom) was dissolved in 4 mL of EG. To obtain the desired Na(acac) starting concentrations the amount of acac^−^ in the precursor was considered and 0.5 M Na(acac) stock solution in EG was diluted with EG to a total volume of 4 mL. The applied Pt concentration of 10.24 mM in 4 mL led to an acac^−^ concentration of around 20.48 mM in Pt(acac)_2_. Therefore, OH^−^ concentrations between 0.047 and 0.5 M were used for the thermal reduction of Pt(acac)_2_ leading to acac^−^/Pt ratios of 4.6 to 48.9, respectively. As the total volume of the added base was kept constant at 4 mL, the reaction mixture was in total 8 mL. The flask was equipped with a reflux condenser. The Pt(acac)_2_ precursor was dissolved at 100 °C for 20 min, resulting in a yellow solution. The mixture was vigorously stirred at 150 °C for 150 min using a preheated oil bath. The precursor solution turned dark brown after about 50 min. After the heating procedure, the reaction mixture was cooled to ambient temperature.

#### 2.1.4. Cleaning of “Surfactant-Free” NPs

Pt NPs were cleaned flocculating the “as-prepared” NP dispersion with two aliquots of 1 M aqueous HCl (VWR, Radnor, PA, USA). The flocculated particles were separated by centrifugation. The supernatant was removed. The particles were washed once by suspending them in 2 aliquots of 1 M aqueous HCl. After a second centrifugation, the supernatant was removed again. The NP were redispersed in acetone for further analysis.

### 2.2. Characterization of Nanoparticles

#### Transmission Electron Spectroscopy (TEM)

TEM was performed with a Tecnai F20 S-Twin Microscope (Fei, Hillsboro, OR, USA) using an acceleration voltage of 200 kV and a magnification of 150 k or with a Jeol 2100 microscope (Jeol Ltd. Akishima, Japan) operated at 200 kV (the latter only for particles synthesized by the reduction of H_2_Pt(OH)_6_ in presence of Na(acac)). For the measurement of liquid NP samples, 0.25 or 0.5 mL of as-prepared NP dispersions were flocculated with 2 aliquots of 1 M HCl and redispersed in 1 aliquots of acetone (see Section 2.1.4). 3 drops of that dispersion were diluted with 1 mL acetone and 3 drops of the diluted acetone dispersions were drop-casted onto the carbon side of the TEM grid (ultrathin Carbon Support Film, Cu 200 mesh, Quantifoil, Großlöbichau, Germany). The averaged particle sizes, the standard deviation, and histograms of the particle sizes distributions were determined using ImageJ counting at least 200 particles.

## 3. Results and Discussion

### 3.1. Thermal Reduction of Pt Precursors with Varying OH^−^ Concentrations

Schrader et al. [13] and Quinson et al. [7] showed the importance of OH^−^ in the reduction of the precursor H_2_PtCl_6_ to obtain stable colloids and to determine the size of NPs in the “surfactant-free” Pt NP synthesis in EG. Recently, the size control by OH^−^ in the reduction of H_2_PtCl_6_ and H_2_PtBr_6_ was used for mechanistic investigations [5]. So far, the size control by OH^−^ in the reduction of H_2_PtCl_6_ was only investigated in the presence of different cations [16] or halides [4,15]. In the present work a halide-free precursor H_2_Pt(OH)_6_ was reduced in presence of NaOH to investigate if in a halide-free system the NP size is still controlled by a certain OH^−^/Pt ratio as reported before [7,13,15]. The solution and reduction step of the precursor H_2_Pt(OH)_6_ used in the present study could not be separated from each other as by heating up the white turbidity changed first to yellow and then brown turbidity before brown dispersions were obtained. Nevertheless, it has been attempted to perform an equivalent procedure to the standard thermal reduction of H_2_PtCl_6_ and H_2_PtBr_6_ [4,13]. H_2_Pt(OH)_6_ and NaOH in EG were stirred at 150 °C in a preheated oil bath adjusting the amount of added NaOH in EG with respect to the amount of OH^−^ of the precursor to obtain the desired OH^−/^Pt ratios (see the experimental section for more details).

**Figure 2:**
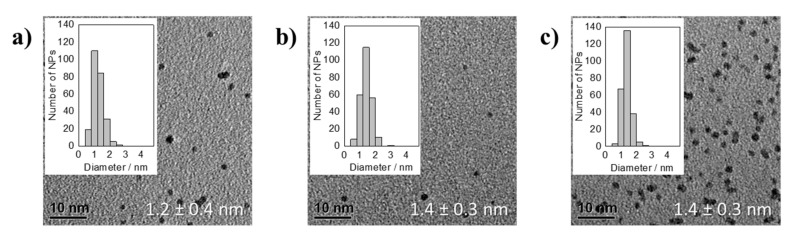
TEM micrographs of averaged particle sizes together with the standard deviation and NP size distributions of thermally formed Pt NPs by using H_2_Pt(OH)_6_ as precursor in presence of NaOH with different OH^−^/Pt ratios of: (**a**) 12.2 (after 10 min of synthesis), (**b**) 9.2 (after 5 min), and (**c**) 7.6 (after 5 min).

The particles obtained in the reduction of H_2_Pt(OH)_6_ in presence of NaOH at a OH^−^/Pt ratio of 48.8 were not stable, they already sintered and as a consequence flocculated after less than 10 min during the synthesis as observed before in the reduction of H_2_PtCl_6_ in presence of NaOH [13]. The reaction mixtures of H_2_Pt(OH)_6_ after 25, 30, 35, and 90 min of synthesis using an OH^−^/Pt ratio of 24.4 showed after 3 days of storage some particle flocculation, but the majority of the NPs remained dispersed in the solution. The NP dispersions with an OH^−^/Pt ratio of 12.2 (after 10, 15, 20, 90 min synthesis time), 9.2, and 7.6 (after 5, 10, 15, 90 min synthesis time) remained stable. The thermal reduction observable by the color change from yellow to brown becomes faster as the OH^−^/Pt ratio decreases (24.4: after about 25 min, 12.2: about 8 min, 9.2: about 1.5 min, 7.6: about 1 min). In the thermal reduction of H_2_PtCl_6_ in the presence of NaOH also at high OH^−^/Pt ratios the color change to dark brown indicating the NP formation occurs after ca. 3 min [7,13]. This different behavior in reduction time implies that in the absence of halides a different mechanism may occur in the polyol process as compared to the one proposed recently [15]. It seems that in the thermal reduction the halide plays a decisive role to initiate the reduction. After a short time of synthesis, when the NPs have been formed indicated by the color change to a dark dispersion, narrow size distributions were obtained. The particle sizes remained the same between the OH^−^/Pt ratios of 7.6 and 9.2, i.e., 1.4 ± 0.3 nm, after 5 min and also for an OH^−^/Pt ratio of 12.2 after 10 min, see Figure 2.

**Figure 3:**
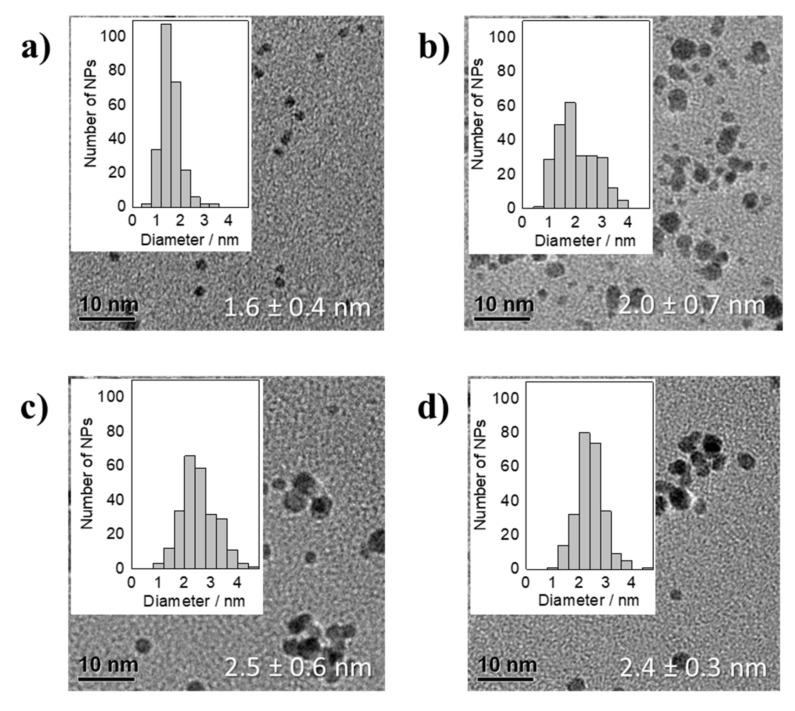
TEM micrographs of averaged particle size together with the standard deviation and NP size distribution of thermally formed Pt NPs by using H_2_Pt(OH)_6_ as precursor in presence of NaOH with different OH^−^/Pt ratios of: (**a**) 24.4, (**b**) 12.2, (**c**) 9.2, and (**d**) 7.6 after 90 min of synthesis.

The particles continued to grow, and quite broad particle size distributions were obtained after 90 min, see Figure 3. At an OH^−^/Pt ratio of 24.4 the particle size of 1.6 ± 0.4 nm is comparable to the other polyol syntheses at an equal OH^−^ starting concentration [4,7,13,15,16]. The particle size increase by lowering the OH^−^ concentration is comparable to previous observations [7,15]. At an OH^−^/Pt ratio of 12.2 the size of 2.0 ± 0.7 nm is however slightly larger as compared to 1.4 ± 0.3 nm (OH^−^/Pt ratio of 12.5) that was described before by Quinson et al. [7] in a chloride containing system. Additionally, at an OH^−^/Pt ratio of 9.2 the particle size remained constant at a diameter of about 2.5 nm while in the chloride containing system with OH^−^/Pt ratios of 6.3 and 10 particle sizes of 2.5 ± 0.3 and 2.1 ± 0.6 nm were reached, respectively [7]. The slightly different particle size together with the larger standard deviation in size could be explained by the lack of chloride that limits the amount of leached Pt species during the reduction and/or the low solubility of H_2_Pt(OH)_6_ in the EG that leads to entangled dissolution and reduction in the NP synthesis.

In summary, at 150 °C in the absence of halides when H_2_Pt(OH)_6_ is used as precursor and NaOH as base, an influence of the OH^−^/Pt ratio to the NP size is shown (see a summary of the size results in Table S1). This behavior is similar to what is observed in the reduction of H_2_PtCl_6_ in presence of NaOH [4,7,13,15,16]. However, the particle sizes in the reduction of H_2_Pt(OH)_6_ are slightly larger as compared to the halide containing systems using H_2_PtCl_6._ Therefore, changing the precursor anion from Cl^−^ to OH^−^ has only a modest effect on the particle size. In contrast, Neumann et al. [4] showed that the precursor anion acac^−^ leads to a substantial size increase in the presence of the base anion OH^−^, as the thermal reduction of the precursor Pt(acac)_2_ in presence of NaOH resulted in particle sizes of ca. 3 nm. This may indicate an influence of the precursor anion within the reduction process. This effect is investigated further in the next section.

### 3.2. Thermal Reduction of Pt Precursors with Varying Acac^−^ Concentrations

In the previous section a slightly different behavior depending on the precursor was shown and hence the precursor anions in the reduction mixture seem to affect the particle size as well. It is now investigated whether a different base anion shows a comparable trend in size control depending on the precursor or if the base anion/Pt molar ratio control the size as it is the case in the previous results. As the precursor Pt(acac)_2_ contains the organic anion acac^−^ that is substantially larger as compared to the inorganic and small OH^−^ anion, Na(acac) was used as base for the reduction of the three precursors H_2_PtCl_6_, H_2_Pt(OH)_6_, Pt(acac)_2_.

**Figure 4:**
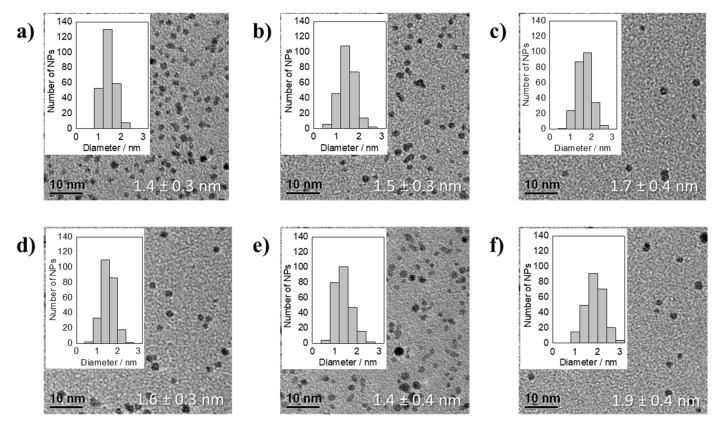
TEM micrographs of averaged particle size together with the standard deviation and NP size distribution of thermally formed Pt NPs by using H_2_PtCl_6_ as precursor in presence of Na(acac) with different acac^−^/Pt ratios of: (**a**) 48.8, (**b**) 24.4, (**c**) 12.2, (**d**) 9.2, (**e**) 6.1, and (**f**) 5.4 after 10 min of synthesis.

By changing the base from inorganic NaOH to organic Na(acac) the thermal reduction of H_2_PtCl_6_ occurred substantially faster (about 1 min of reduction) and heating periods longer than 20 min led to sintering and flocculation of the NPs. This is different to the previous experiments. The presence of acac^−^ and chloride anions seem not to be able to take over the stabilizing role that OH^−^ fulfills in the experiments using H_2_PtCl_6_ or H_2_PtBr_6_ as precursors. Therefore, it can be assumed that the NPs might be less protected against an attack by chloride resulting in Ostwald ripening as discussed by Neumann et al. [4] or coalescence. While the NPs sintered during longer reduction times, after 10 min the acac^−^/Pt ratios of 48.8, 24.4, 12.2, 9.2, 6.1 and 5.4 led to stable NPs with sizes within the size error of 1.4–1.9 nm determined by TEM analysis, see Figure 4. The obtained particle sizes in Figure 4 were slightly smaller at low OH^−^/Pt as compared to the size reported for the reduction of H_2_PtCl_6_ in presence of NaOH [7]. Using an acac^−^/Pt ratio of 4.6 and 2.4 did not lead to stable NPs. The NPs already sintered during the thermal reduction, but at the higher acac^−^ concentrations the dispersions remained stable for several days. Interestingly, although the reduction rate and stability are affected using Na(acac), in the reduction of H_2_PtCl_6_ the particle size was comparable to the system with NaOH as base and no Ostwald ripening was observed. Hence, the smaller NPs might be obtained due to leaching induced by chloride without a following Ostwald ripening, which was observed also by Neumann et al. [4] only after longer reaction times.

**Figure 5:**
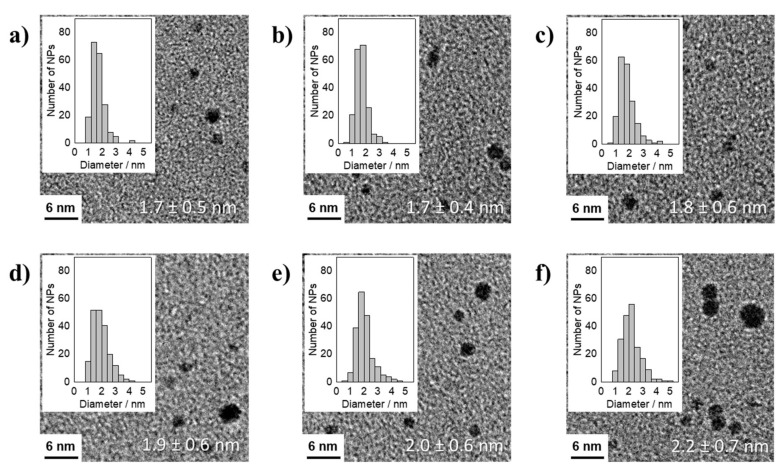
TEM micrographs of averaged particle size together with the standard deviation and NP size distributions of thermally formed Pt NPs by using H_2_Pt(OH)_6_ as precursor in presence of Na(acac) with different acac^−^/Pt ratios of: (**a**) 48.8, (**b**) 24.4, (**c**) 12.2, (**d**) 9.2, (**e**) 6.1, and (**f**) 4.6 after 90 min of synthesis.

The reduction of H_2_Pt(OH)_6_ in presence of Na(acac) led to stable particles after 90 min at 150 °C between acac^−^/Pt ratios of 4.6 and 48.8. At acac^−^/Pt ratios of 28.8 and 24.4 the particle size was constant at 1.7 ± 0.5 and 1.7 ± 0.4 nm, respectively, see Figure 5. Decreasing the acac^−^/Pt ratio led to an increase in size to 2.2 ± 0.7 nm at an acac^−^/Pt ratio of 4.6. The size increase in Figure 5 was comparable to the reduction of H_2_Pt(OH)_6_ in presence of NaOH, see Figure 3, but the results suggest that slightly smaller particle sizes are reached. In conclusion, the presence of Na(acac) in the reduction of H_2_Pt(OH)_6_ leads to slightly smaller particles in comparison to the use of NaOH, compare Figure 5 to Figure 3. As compared to the reduction of H_2_PtCl_6_ no chloride was present that could induce leaching. Hence, the smaller particles must be explained by an interaction of acac^−^ and OH^−^ as “ligands” stabilizing the Pt NP surface against coalescence.

**Figure 6:**
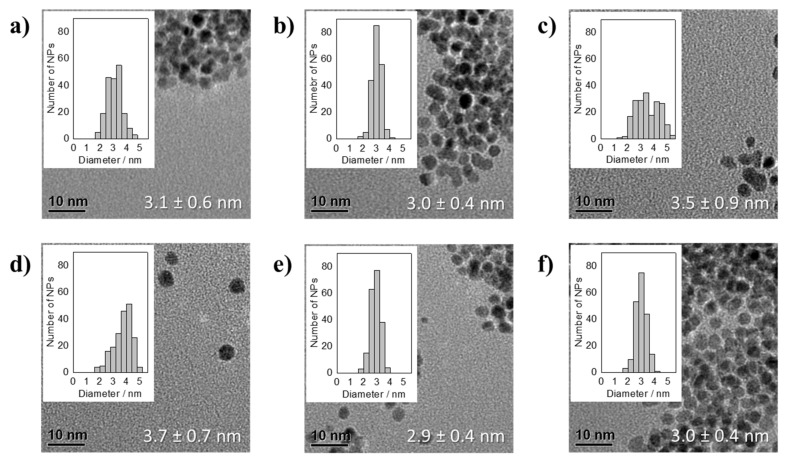
TEM micrographs of averaged particle size together with the standard deviation and NP size distribution of thermally formed Pt NPs by using Pt(acac)_2_ as precursor in presence of Na(acac) with different acac^−^/Pt ratios of: (**a**) 24.4, (**b**) 12.2, (**c**) 9.1, (**d**) 6.1, (**e**) 5.4, and (**f**) 4.6 after 150 min of synthesis.

Reducing Pt(acac)_2_ in the presence of Na(acac) leads after 150 min of synthesis to NP dispersions that remained stable for several hours, but the TEM analysis showed a fast particles agglomeration, see Figure 6. In addition, after one day in most samples a yellow to light brown solid was found depositing on the wall and the bottom of the glass vials. The solid is probably an organic based compound that might contain acac^−^ and precipitates as it is not anymore soluble in EG at room temperature. The acac^−^/Pt ratio of 48.9 did not lead to stable particles. The NP size at an acac^−^/Pt ratio of 24.4 was 3.1 ± 0.6 nm, see Figure 6. In the range of an acac^−^/Pt ratio between 4.6 and 24.4 the particle size varied between 2.9 ± 0.4 and 3.7 ± 0.7 nm at an acac^−^/Pt ratio of 5.4 and 6.1, respectively. At a particle size of above 3 nm, NPs also synthesized by H_2_PtCl_6_ in presence of NaOH by Quinson et al. [7] showed aggregation on the TEM grid. Interestingly, to reach such sizes substantially lower OH^−^/Pt ratios of 5.5 or lower were necessary [7]. This might be explainable by the substantially larger size of the acac− as compared to the OH^−^ anion, as less acac^−^ can be located on the Pt NP surface due to sterically hindering of the “ligands”. Hence, even at higher acac^−^ concentration substantially less anions are needed to completely saturate the surface resulting in less negative charge located on the surface. Due to the less negative charge density the Coulomb repulsion between the NPs is reduced, which increases the probability of agglomeration.

The size of the particles obtained from the reduction of Pt(acac)_2_ in presence of Na(acac) are consistent with the sizes obtained by Neumann et al. [4] of 3.0 ± 0.3 nm for reducing Pt(acac)_2_ in presence of NaOH as base in EG. Consequently, it seems that it is not the presence of acac^−^ but the use of the Pt(acac)_2_ precursor that leads to a substantial particle increase. Considering the previous experiments, the base anion seems to be less important for the particle size but mainly the nature of the precursor (oxidation state, bonding strength, etc.) determines the size control. The nature of the precursor itself would be expected to be only important in the first step of the reduction mechanism. Assuming a size control determined by the Pt salt could be explained by the following: The Pt precursor influences the reduction rate, i.e., how fast the coordinated Pt can be reduced or how fast the “ligands” are exchanged with base anions. The latter depends on the ligand properties and in particular on the binding properties of the discussed anions (OH^−^, acac^−^, or chloride) to Pt. This is important for the (further) reduction and growth process as the precursor anions are expected to be located or to be bond to the surface during the particle formation. Small precursor anions (OH^−^) lead to smaller particles, see Figure 3 to Figure 5, while larger precursor anions (acac^−^) lead to larger particles, see Figure 6 and a summary of the size results in Table S1. Using larger anions, less “ligands” can be located at or adsorbed to the Pt surface. As a result, less charge is present at the particle surface to stabilize even at higher anion concentrations thus larger NPs are formed. As the use of Pt(acac)_2_ independent of the amount of NaOH or Na(acac) led to the same large particles, the sterically demanding acac^−^ (from the precursor) seems to bind strongly to the Pt, determining the size independent of other anions present within the reaction medium. Therefore, the size determining step seems to be the shielding of the acac^−^ limiting the number of bound acac^−^ “ligands” to the Pt, hence leading to a size increase, or, e.g., hindering the “ligand” exchange by OH^−^ when NaOH is used as base.

## 4. Conclusions

Comparison of the NP sizes obtained in thermal reduction of the precursors H_2_PtCl_6_, H_2_Pt(OH)_6_, and Pt(acac)_2_ in presence of NaOH or Na(acac) in EG revealed that the nature of base anions does not substantially influence the size. Instead, the precursor anion or the nature of the metal salt seem to play a more important role in the particle size control. Interestingly, the thermal reduction of Pt(acac)_2_ leads to large particles of about 3 nm independent of the NaOH or Na(acac) concentration in EG, while the size control of H_2_PtCl_6_ and H_2_Pt(OH)_6_ depends on the OH^−^/Pt or acac^−^/Pt ratio.

## Data Availability

The data presented in this study are available on request from the corresponding author.

## Supplementary Materials

The following are available online at https://www.mdpi.com/article/10.3390/nano11082092/s1. Table S1. Overview of the averaged particle sizes and standard deviations determined from TEM images reducing different precursors in presence of the bases NaOH and Na(acac) depending on the ratio of the anion of the base to Pt. * When the precursor contains the same anion as the base the amount is considered in the anion/Pt ratio. # Some base anion/Pt ratios led to instable particles.
