# Supplementary material for: Anion Dependent Particle Size Control of Platinum Nanoparticles Synthesized in Ethylene Glycol
Source: Nanomaterials (Basel). 2021 Aug 18;11(8):2092. doi: 10.3390/nano11082092 (PMC8400561; doi:10.3390/nano11082092)
Supplement: Supplementary file 1 [file nanomaterials-11-02092-s001.zip › nanomaterials-1325808-supplementary.pdf]

Supplementary Materials

Communication

Anion Dependent Particle Size Control of Platinum Nanoparticles Synthesized in Ethylene Glycol

Johanna Schröder [1,2,*], Sarah Neumann [1], Jonathan Quinson [3], Matthias Arenz [2] and Sebastian Kunz [1,4,*]

[1] Institute of Applied and Physical Chemistry (IAPC), Center for Environmental Research and
Sustainable Technology, University of Bremen, Leobener Strasse 6, 28359 Bremen, Germany
[2] Department of Chemistry, Biochemistry, and Pharmaceutical Sciences (DCBP), University of Bern,
Freiestrasse 3, 3012 Bern, Switzerland
[3] Chemistry Department, University of Copenhagen, Universitetsparken 5, 2100 Copenhagen, Denmark;
[4] Südzucker AG, Central Department Research, Development, and Services (CRDS), Wormser Strasse 11, 67283 Obrigheim, Germany
* Correspondence: johanna.schroeder@unibe.ch (J.S.); sebastian-kunz@web.de (S.K.)

Table S1. Overview of the averaged particle sizes and standard deviations determined from TEM images reducing different precursors in presence of the bases NaOH and Na(acac) depending on the ratio of the anion of the base to Pt. * When the precursor contains the same anion as the base the amount is considered in the anion/Pt ratio. # Some base anion/Pt ratios led to instable particles.

**Precursor**		**$H_2Pt(OH)_6$**			**H_2PtCl_6**	**$Pt(acac)_2$**
**Base**		**NaOH ***		**Na(acac)**	**Na(acac)**	**Na(acac) ***
Reduction time		10 min	90 min	90 min	10 min	150 min
	48.8	-	-	1.7 ± 0.5	1.4 ± 0.3	- #
	24.4	- #	1.6 ± 0.4	1.7 ± 0.4	1.5 ± 0.3	3.1 ± 0.6
base	12.2	1.2 ± 0.4	2.0 ± 0.7	1.8 ± 0.6	1.7 ± 0.4	3.0 ± 0.4
anion/	9.2	1.4 ± 0.3	2.5 ± 0.6	1.9 ± 0.5	1.6 ± 0.3	3.5 ± 0.9
Pt	7.6	1.4 ± 0.3	2.4 ± 0.3	-	-	-
molar	6.1	-	-	2.0 ± 0.6	1.4 ± 0.4	3.7 ± 0.7
ratio *	5.4	-	-	2.2 ± 0.7	-	2.9 ± 0.4
	4.6	-	-	- #	1.9 ± 0.4	3.0 ± 0.4
	2.4	-	-	- #	-	-
